# Creative Self-Efficacy, Academic Performance and the 5Cs of Positive Youth Development in Spanish Undergraduates

**DOI:** 10.3390/jintelligence13090120

**Published:** 2025-09-17

**Authors:** Diego Gomez-Baya, Francisco Jose Garcia-Moro, Gina Tomé, Margarida Gaspar de Matos

**Affiliations:** 1 Department of Social, Developmental and Educational Psychology, Universidad de Huelva, 21071 Huelva, Spain; fjose.garcia@dpsi.uhu.es; 2 School of Health Sciences, Universidade Europeia, 1500-210 Lisboa, Portugal; gina.tome@universidadeeuropeia.pt; 3 School of Human Sciences, Universidade Católica Portuguesa, 1649-023 Lisboa, Portugal; mmmatos@ucp.pt

**Keywords:** creative self-efficacy, positive youth development, performance, higher education, youth

## Abstract

(1) Background: Creative self-efficacy is associated with better psychological well-being and academic performance in adolescent and youth samples. Positive youth development is a strength-based model of youth transition to adulthood, which states that this emerges from adaptive regulations between personal strengths and nurturing contexts. The present study aimed to examine the associations between creative self-efficacy, PYD and perceived academic performance in a sample of Spanish youth. (2) Methods: A cross-sectional study was carried out during the spring of 2024. A sample composed of 370 undergraduates (M = 21.29, SD = 3.61) from 10 universities in Andalusia (Spain) filled in an online self-report measure. (3) Results: The results showed positive associations between creative self-efficacy, PYD and academic performance. A mediational analysis indicated that creative self-efficacy presented a positive effect on perceived academic performance through its positive associations with both Confidence and Competence dimensions of PYD. (4) Conclusions: These results may suggest the need to integrate creativity and PYD programs to strengthen academic performance in higher education.

## 1. Introduction

Creativity is defined as the tendency to produce original and effective ideas (Runco and Jaeger 2012). Literature has concluded that creativity is associated with positive outcomes in human development, such as intelligence development, academic performance and subjective well-being (Du et al. 2020). Recently, researchers have also addressed the individual and contextual factors associated with creativity, such as personality, cognitive style or supportive context (Alfaro Muirhead et al. 2024; Asquith et al. 2022; Park et al. 2023). Among the psychological mechanisms underlying creative behavior, creative self-efficacy refers to the belief in one’s own ability to produce creative outcomes (Caballero et al. 2024; Tierney and Farmer 2002). This concept is derived from Bandura’s Social Cognitive Theory, which stated that self-efficacy plays a key motivational role in the process of creativity and innovation (Bandura 1997; Puente-Díaz 2016).

Thus, creative self-efficacy is an important antecedent of creative performance, because creative behavior requires effort and persistence in the face of potential obstacles (Tierney and Farmer 2002). Having a high level of creative self-efficacy can facilitate concentration and improve self-confidence when tackling a task (Du et al. 2020). Moreover, the development of creative self-efficacy is associated with creative role identity and perceived creative expectation (Tierney and Farmer 2011). In this line, a recent meta-analysis by Haase et al. (2018) concluded that creative self-efficacy was positively associated with various measures of creativity. The effect size was smaller for objective measures of creative performance and stronger for self-report measures. Furthermore, creative self-efficacy has been identified as a mechanism between creative mindsets and creative problem solving (Mathisen and Bronnick 2009; Royston and Reiter-Palmon 2019). Educational practices should provide opportunities to enjoy diverse experiences, perspectives and knowledge to reinforce the use of creative problem-solving skills (Tierney and Farmer 2002).

In youth and adolescent samples, literature has also indicated that creative self-efficacy is associated with better psychological well-being and academic performance. Concerning youth samples, two studies have been developed in China and the US. In a sample of Chinese undergraduates, Fino and Sun (2022) found that the personality traits of openness to experience and conscientiousness were related to more wellbeing through its impact on creative self-efficacy. Furthermore, in US university students, Stolz et al. (2022) concluded that the improvement in creative self-efficacy was linked to better coping with current academic and future career challenges. With regards to adolescent samples, other two works, from the US and Poland, have provided some evidence about the correlates of creative self-efficacy in academic and psychological adjustment. In adolescents from the USA, Beghetto (2006) found that students with more creative self-efficacy reported more positive beliefs about their academic abilities in all subjects, expressed higher intention to enroll in college and demonstrated a higher engagement in after-school extracurricular activities. Moreover, concerning well-being, Karwowski et al. (2018) found positive interrelations between creative self-efficacy, self-esteem and emotional intelligence in a sample of Polish adolescents.

The connections between creative self-efficacy, psychological well-being and academic outcomes could be well understood within the Relational Developmental Systems Theory (RDST; Overton 2013). RDST is a meta-theory in developmental science which conceptualizes living organisms as active agents within their contexts. Integrated into RDST, Positive Youth Development (PYD) is a strength-based model of youth transition to adulthood, which states that positive outcomes in development emerge from the adaptive regulations between personal strengths and nurturing contexts (Lerner et al. 2011). In recent decades, research across countries has supported the 5Cs model of PYD (Dimitrova and Wiium 2021). According to Lerner et al. (2021), these 5Cs are as follows: Competence (refers to a sense of efficacy in different life areas), Confidence (i.e., positive self-image and self-esteem), Connection (involves strong and positive relationships with others), Character (the internalization of social and cultural norms) and Caring (represents empathy and compassion toward others). When these five dimensions are fulfilled, a sixth one—called Contribution—emerges. This Contribution includes meaningful social engagement that benefits the individual, family, peers and civil society. These 5Cs serve as thriving indicators because they are linked to better physical health and psychological well-being in youth (Lewin-Bizan et al. 2010). Also, they act as protective factors against risky behaviors, such as substance use, delinquency and emotional difficulties (Lerner et al. 2014).

Complementing the PYD model and rooted in the RDST, Benson (2007) introduced the model of Developmental Assets. This model describes the key conditions that support PYD. These assets or resources are divided into two groups: internal assets (i.e., commitment to learning, positive values, social competences and positive identity) and external assets (e.g., social support, empowerment, clear boundaries and expectations, and constructive use of time). In this line, creative self-efficacy may be integrated into the group of internal assets to promote PYD, since it implies self-regulated cognitive skills to facilitate knowledge construction, task completion, problem solving and decision making (Sun and Hui 2012). Specifically, creative self-efficacy is well-connected with positive identity (Karwowski et al. 2018) and commitment to learning assets (Gu et al. 2017). Creative self-efficacy is conceived as a protective factor at individual level that may influence the emergence of positive outcomes in youth development (Puente-Diaz and Cavazos-Arroyo 2018), jointly with some contextual supportive conditions (Nemeržitski and Heinla 2020).

Concerning the links between PYD and academic achievement, some works have provided some supportive evidence. From developmental assets framework, Beck and Wiium (2019) found in a sample of Norwegian high school students that some developmental assets (i.e., commitment to learning, support and positive identity) were positively connected with academic achievement. In a study with Slovenian adolescents, Kozina et al. (2019) concluded that Character and Confidence dimensions of PYD were associated with better math achievement. Finally, Abdul Kadir et al. (2021) included self-reported creativity as a part of PYD measure, including a seventh C. Creativity was found to be correlated with the five original Cs of PYD and to have a positive effect on mindfulness skills. Thus, creative self-efficacy may fit very well into this RDST, as an internal asset that may be associated with more PYD and in turn with positive academic outcomes in youth.

### The Present Study

Most PYD research to date has addressed the connections between PYD, risk behaviors and mental health, while further work is still needed to examine academic outcomes. As far as we know, no study to date has explore this connection in Spanish youth samples. Moreover, despite the research evidence for the positive interrelations between creative self-efficacy, wellbeing and academic outcomes, no study has examined these relationships within the framework of the 5Cs of PYD. Following RDST model, creative self-efficacy, as an internal asset, could be related to better academic achievement through improvements in PYD. Thus, the present work aimed at examining the associations between creative self-efficacy, PYD and perceived academic performance in a sample of Spanish youth. We expected positive associations between creative self-efficacy, PYD and academic performance, in line with previous literature. Specifically, as a main expected contribution, the present work aimed at exploring the mediation of the 5Cs of PYD in the relationship between creative self-efficacy and perceived academic performance.

## 2. Materials and Methods

### 2.1. Data Collection Procedure and Sample Composition

A cross-sectional study was conducted during the spring of 2024 using an online self-report questionnaire. Undergraduate students completed the survey in approximately 30 min. The research adhered to the ethical standards established in the Declaration of Helsinki and was approved by the Institutional Review Board of University of Huelva on 10 January 2019 (UHU1259711). Participation was entirely voluntary, with no financial compensation offered, and all participants provided written informed consent.

In total, 370 undergraduate students participated (67.2% women, 31.4% men and 1.4% non-binary), ranging in age from 18 to 29 years old (M = 21.29, SD = 3.61). They were enrolled in 10 universities across Andalusia, a region in southern Spain: the Universities of Almería, Cádiz, Córdoba, Granada, Huelva, Jaén, Málaga, Seville, Pablo de Olavide University (Seville) and Loyola University (Seville and Córdoba). Most participants lived in cities with populations above 300,000 (38.4%) or between 50,001 and 300,000 (31.1%). Most people lived with their parents (49.5%) or with roommates (30.5%).

More than half (54.5%) reported that they were not in a romantic relationship, and 65.6% of the participants were not currently seeking employment. Regarding academic disciplines, 39.7% of the participants were studying degrees in Law or Social Sciences, 29.6% in Sciences or Engineering, 19.2% in Arts and Humanities and 11.5% in Health Sciences. About half of the students were in their first or second year of study, 42.6% were in their third year and 7.4% were in their fourth year or higher.

### 2.2. Instrument

Positive youth development. The short form of the Positive Youth Development (PYD) scale developed by Geldhof et al. (2014) was used in this study. A Spanish adaptation by [Anonymized] was utilized, showing strong internal consistency and factorial validity. The instrument consists of 34 items distributed across five dimensions, representing the 5Cs: Competence (6 items, e.g., “I have a lot of friends”); Confidence (6 items, e.g., “I like my physical appearance”); Character (8 items, e.g., “I never do things I know I shouldn’t do”); Connection (8 items, e.g., “I am a useful and important member of my family”) and Caring (6 items, e.g., “It bothers me when bad things happen to other people”). Responses were recorded using a 5-point Likert scale, with formats varying by item (e.g., from 1 = “Not at all important” to 5 = “Very important”; 1 = “Strongly disagree” to 5 = “Strongly agree”; 1 = “Not at all” to 5 = “Very much” or 1 = “Never or almost never” to 5 = “Always”). The reliability coefficients for each dimension were acceptable: Character (α = 0.66, Ω = 0.66), Competence (α = 0.67, Ω = 0.69), Confidence (α = 0.74, Ω = 0.77), Connection (α = 0.75, Ω = 0.75) and Caring (α = 0.79, Ω = 0.78).

Creative self-efficacy. The Creative Self-efficacy Scale, developed by Yi (2008) and adapted to Spanish by Aranguren and Irrazabal (2011), was administered. This scale is composed of five items (“I am certain that I can produce novel and appropriate ideas” or “When I am confronted with a problem, I can try several solutions to solve it”), with a 4-point Likert response, ranging from “not at all true” to “exactly true”. Acceptable internal consistency reliability was observed, with α = 0.77 and Ω = 0.77.

Perceived performance at the university. This variable was evaluated by using this question “How is your academic performance?”. Five response options were showed to be selected: 1 = “Low”, 2 = “Sufficient”, 3 = “Good”, 4 = “Very good” and 5 = “Excellent”).

### 2.3. Data Analysis Design

The Kolmogorov–Smirnov normality test showed that study variables were non-normally distributed and non-parametric and robust analyses were used. First, descriptive statistics were examined for the 5Cs of PYD, creative self-efficacy and perceived academic performance. Second, bivariate Spearman correlations were conducted among study variables. Third, two hierarchical regression analyses were separately performed to explain creative self-efficacy, based on demographics (i.e., gender and age) and the 5Cs of PYD, and to explain perceived academic performance, based on demographics, creative self-efficacy and the 5Cs. Standardized coefficients, 95% Confidence intervals, R-squared and Durbin–Watson score were described in these regression analyses, conducted with SPSS 21.0. Fourth, based on the previous results, some mediation models were tested to explore the mediation role of each dimension of PYD in the relationship between creative self-efficacy and perceived academic performance. Total, direct and indirect effects were detailed, as well as Z scores and bootstrapping confidence intervals. These regression-based mediational models were designed and tested with JASP 0.18.3.0., according to the indications by Hayes (2013) and Baron and Kenny (1986). Finally, based on the previous results from the mediational analyses, a structural equation model was tested using statistical package EQS 6.1, to integrate in a confirmatory model the relationships observed. Standardized coefficients were reported, and robust fit indicators, such as Satorra–Bentler χ^2^, CFI, NNFI and RMSEA, were calculated to examine overall data fit. These statistics were interpreted following indications by Byrne (2013).

## 3. Results

### 3.1. Descriptive Statistics and Bivariate Correlations

Table 1 shows the descriptive statistics and Spearman bivariate correlations among study variables. The results indicated moderate high scores in creative self-efficacy and self-perceived academic performance. Up to 86.6% of the participants indicated positive academic performance, reporting good (44.8%), very good (34.1%) or excellent performance (7.7%). Regarding positive youth development, the highest mean scores were found in caring and character, while the lowest one was detected in competence. Furthermore, correlation analyses showed positive associations between creative self-efficacy, the 5Cs of PYD and perceived performance. The strongest associations with creative self-efficacy were found with confidence, character and competence. Moreover, the strongest correlations with academic performance were detected with competence and confidence.

### 3.2. Regression and Mediation Analyses

Table 2 and Table 3 describe the results of two hierarchical regression analyses to respectively explain the creative self-efficacy, based on demographics (gender and age) and the 5Cs of PYD, and to explain academic performance based on demographics, creative self-efficacy and the 5Cs of PYD. Concerning creative self-efficacy, gender had a small positive effect, t(358) = −1.99, *p* = .047, d = −0.23, with men showing a higher mean (M = 3.07, SD = 0.51) than women (M = 2.96, SD = 0.50). In the second step, confidence and competence showed positive effects to explain creative self-efficacy in nearly 20% (Durbin–Watson = 2.07). Furthermore, creative self-efficacy, competence and confidence were found to present positive effects to explain academic performance (R^2^ = 0.117), with no significant effects by demographics. No autocorrelation in the residuals of the regression analysis was observed, since Durbin–Watson = 1.86.

**Figure 1:**
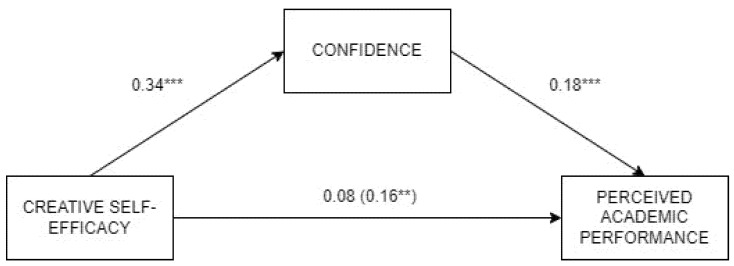
Analysis of the mediation by Confidence in the relationship between creative self-efficacy and perceived academic performance. Note: *** *p* < .001, ** *p* < .01.

Based on the preliminary results, two mediational models were tested to examine the mediational role of competence and confidence in the relationship between creative self-efficacy and academic performance (Table 4). The total effect by creative self-efficacy on perceived performance was significant (β = 0.16, *p* = .003, LLCI = 0.06, ULCI = 0.26), before adding the mediators. First, confidence was found to totally mediate the relationship between creative self-efficacy and academic performance, so that high creative self-efficacy was associated with high confidence, and in turn with high performance (Figure 1). After including the mediator, the direct effect by creative self-efficacy was not significant. Thus, a small positive indirect effect (β = 0.07, *p* < .001, LLCI = 0.03, ULCI = 0.12) was observed through the mediation by confidence. The model achieved a reduced explained variance of 6.5%.

**Figure 2:**
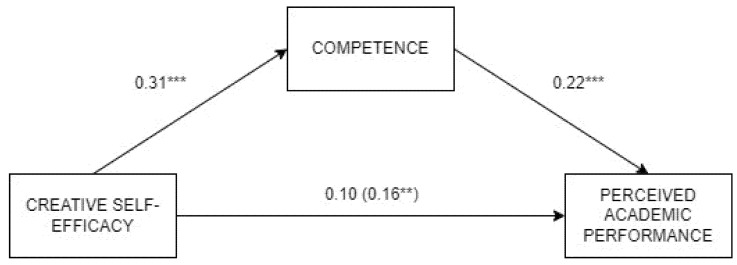
Analysis of the mediation by Competence in the relationship between creative self-efficacy and perceived academic performance. Note: *** *p* < .001, ** *p* < .01.

Second, competence was also found to totally mediate the link between creative self-efficacy and performance (Figure 2). The direct effect by creative self-efficacy on performance was not significant after including competence as mediator, which showed a small positive indirect effect (β = 0.06, *p*= .003, LLCI = 0.02, ULCI = 0.09). Thus, creative self-efficacy had a positive effect on competence, and in turn competence was positively related to perceived performance. The model achieved a reduced explained variance of 5.5%.

### 3.3. Structural Equation Model

**Figure 3:**
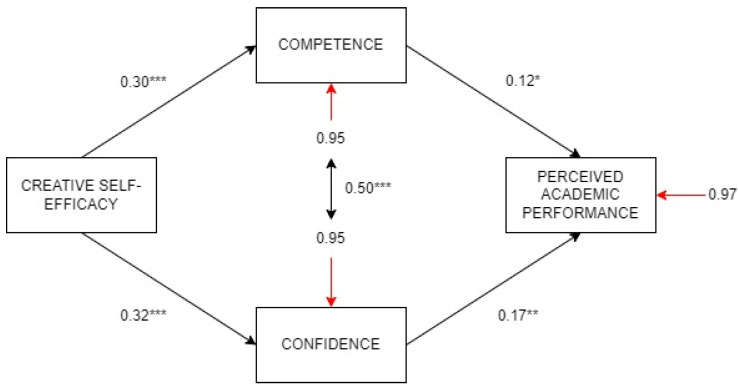
Structural equation modeling of the multiple mediation of Competence and Confidence in the relationship between creative self-efficacy and perceived academic performance. Note: *** *p* < .001, ** *p* < .01, * *p* < .05.

Figure 3 shows the confirmatory model tested, which integrated the multiple mediation by competence and confidence in the relationship between creative self-efficacy and perceived academic performance. Standardized coefficients are presented in the relationships between variables. The model reached good overall data fit, Satorra–Bentler χ^2^ = 1.42, *p* = .233, CFI = 0.997, NNFI = 0.983 and RMSEA = 0.034. Concerning explained variance, R^2^ = 0.066 was observed for perceived performance. Competence reached R^2^ = 0.090, and confidence, R^2^ = 0.105. In this model, creative self-efficacy had medium-sized positive effects on competence and confidence, and these two mediators had small-sized positive effects on perceived academic performance. Competence and confidence were positively interrelated, showing a big size effect. Thus, the two dimensions of PYD were total mediators between creative self-efficacy and perceived academic performance.

## 4. Discussion

The main aim of the present work was to analyze the associations between creative self-efficacy, PYD and perceived academic performance. The results showed that the 5Cs of PYD presented positive associations with both creative self-efficacy and perceived academic performance. Concretely, Competence and Confidence had the strongest effects on perceived performance, after controlling creative self-efficacy. The results underlined the role of creative self-efficacy as an internal asset for PYD, consistently with RDST (Overton 2013), and with the previous literature about the connections between creative self-efficacy and well-being, such as Fino and Sun (2022) and Karwowski et al. (2018). The main contribution of the present work was the analysis of the mediation by Competence and Confidence in the relationship between creative self-efficacy and perceived academic performance. Our results pointed out that these two dimensions of PYD were total mediators in that association, so that creative self-efficacy was positively related to both competence and confidence, and these Cs were positively associated with greater perceived academic performance. Despite the significant results observed, the indirect effects had a small size, and the model’s explained variance was low. This mediational role of PYD is consistent with previous research about positive academic outcomes derived from PYD, such as the works by Beck and Wiium (2019) and Kozina et al. (2019). Specifically, the dimensions of Confidence and Competence were the Cs most strongly associated with creative self-efficacy, because they respectively referred to positive self-worth and general self-efficacy. Thus, the present research provides new evidence for the application of the RDST to the relationship between creative self-efficacy and academic performance, mediated by PYD. High creative self-efficacy may encourage young people to make greater efforts to cope with academic challenges by fostering their self-esteem and general self-efficacy. In turn, better academic achievement emerged from both higher creative self-efficacy and well-being. The present work observed some small gender differences in creative self-efficacy, with men reporting higher scores than women. Despite the evidence by Baer and Kaufman (2008) concluding the lack of consistent gender differences in creativity, this result is consistent with research on gender differences in self-efficacy and self-perceived skills (Gómez-Baya et al. 2017; Wismath and Zhong 2014).

Some practical implications may be derived from these contributions. Interventions to foster creative self-efficacy in higher education jointly with PYD should be encouraged to improve academic achievement (Alt et al. 2023). The importance of creative cognitive processes in higher education has been well recognized (Miller and Dumford 2016). Some authors have emphasized the importance of creating environments that foster creativity in higher education institutions, for example, by having enough time and space for creativity development, presenting varied educational situations, allowing students to work in new ways, challenging them with real problems, taking into account their previous knowledge, encouraging them to pursue topics that interest them most and understanding their individual differences in problem solving (Mathisen and Bronnick 2009; Soriano de Alencar et al. 2017). In this vein, some programs have received empirical support. In China, Byrge and Tang (2015) developed an embodied creativity training program for undergraduates, based on creative fitness exercising, 20 h workshop about creative techniques, a national entrepreneurship festival and a final theoretical reflection. This program was found to increase creative self-efficacy and creative production. Furthermore, in Norway, Mathisen and Bronnick (2009) conducted a program for students and municipality employees with lectures, discussions and demonstrations about central theories and research on creativity. Self-efficacy levels increased significantly for both students and municipality employees. Another intervention in a Portuguese university found that a cooperative learning program to teach creative skills improved creative and divergent thinking (Catarino et al. 2019). A recent experience conducted with higher education students in Israel found that a 3-month Future Problem Solving program focused on peace education and teacher training through engendering creativity and innovation skills improved their beliefs about their abilities to produce creative ideas and innovative behaviors. These creativity programs may be well-integrated into PYD promotion programs in university context, such as the 4 h program (Arnold and Gagnon 2020; Lerner et al. 2014), which was found to be effective in promoting good academic outcomes, such as increased academic competence and engagement (Li et al. 2008; Ma et al. 2009).

Despite the contributions and potential implications for practice, some limitations may be acknowledged in the conclusions of this study. First, the conclusions are only based on associations between the variables and directionality cannot be concluded. The directionality in the relationships included in the model are only inferences based on regression analyses. The examination of causal relationships requires an experimental design, while the establishment of directionality needs a longitudinal design. Concerning the assessment of creativity, the present work addressed the analysis of creative self-efficacy, which could be well measured by using a self-report. Other instruments may complete the study of creativity by administering other instruments, such as Kaufman Domains of Creativity Scale (Kaufman 2012), Creative Potential and Practised Creativity (DiLiello and Houghton 2008) or Lifetime Creativity Scales (Richards et al. 1988). Moreover, the measurement of perceived academic performance relies solely on a single self-reported item, which may reduce the validity of the outcome variable. The use of more comprehensive and validated instruments used in prior research is recommended, integrating the assessment of academic performance from multiple dimensions. As well, the regression models reached a small-sized explained variance for perceived academic performance. This result indicates that PYD dimensions and creative self-efficacy, although significant, had small effects on perceived academic performance, and other variables should be included in the regression equations. Thus, future research may also examine other educational predictors to increase explanatory power of the model, such as academic self-efficacy, academic anxiety, motivation and contextual factors (Clark et al. 2014; Klomegah 2007; Shakir 2014). Another limitation stemmed from the size and characteristics of the sample. The use of a small and convenient sample limits the generalization of the results to the undergraduates’ population in Spain.

## 5. Conclusions

In conclusion, this manuscript provides evidence for the positive associations between creative self-efficacy, PYD and academic performance. Creative self-efficacy was found to have a positive effect on perceived academic performance through its positive associations with both Confidence and Competence dimensions of PYD. These results may suggest the need to integrate creativity and PYD programs in Higher education to strengthen academic performance.

## Figures and Tables

**Table 1:** Descriptive statistics and bivariate correlations.

	M	SD	1	2	3	4	5	6	7
1.-Creative self-efficacy	2.99	0.51	(1)						
2.-Academic performance	3.34	0.86	0.15 **	(1)					
3.-Character	3.87	0.47	0.38 ***	0.19 ***	(1)				
4.-Competence	2.86	0.66	0.30 ***	0.17 **	0.22 ***	(1)			
5.-Confidence	3.68	0.65	0.36 ***	0.20 ***	0.40 ***	0.54 ***	(1)		
6.-Caring	4.17	0.59	0.17 **	0.18 ***	0.39 ***	−0.01	0.02	(1)	
7.-Connection	3.52	0.63	0.22 ***	0.20 ***	0.40 ***	0.35 ***	0.42 ***	0.13 *	(1)

Note. *** *p* < .001, ** *p* < .01, * *p* < .05.

**Table 2:** Hierarchical regression analysis to explain creative self-efficacy.

DV: Creative Self-Efficacy				
	F/R^2^	β	LLCI	ULCI
Step 1	2.48/0.014			
Gender		0.10 *	0.01	0.45
Age		0.06	−0.06	0.23
Step 2	12.41 ***/0.199			
Gender		0.11 *	0.02	0.45
Age		0.11 *	0.02	0.28
Character		0.19 **	0.06	0.31
Competence		0.15 *	0.04	0.27
Confidence		0.18 **	0.05	0.31
Caring		0.07	−0.04	0.18
Connection		0.01	−0.11	0.112

Note. *** *p* < .001, ** *p* < .01, * *p* < .05. DV = Dependent variable.

**Table 3:** Hierarchical regression analysis to explain perceived performance.

DV: Academic Performance				
	F/R^2^	β	LLCI	ULCI
Step 1	1.94/0.011			
Gender		−0.10	−0.44	0.01
Age		0.02	−0.12	0.18
Step 2	4.95 **/0.041			
Gender		−0.12 *	−0.48	−0.03
Age		0.01	−0.13	0.16
Creative self-efficacy		0.18 **	0.07	0.28
Step 3	5.64 ***/0.117			
Gender		−0.10	−0.44	0.02
Age		0.05	−0.08	0.21
Creative self-efficacy		0.06	−0.05	0.17
Character		−0.01	−0.14	0.12
Competence		0.13 *	0.01	0.26
Confidence		0.14 *	0.01	0.28
Caring		0.10	−0.01	0.22
Connection		0.08	−0.04	0.20

Note. *** *p* < .001, ** *p* < .01, * *p* < .05. DV = dependent variable.

**Table 4:** Mediational analyses.

CONF MEDIATION	PERF R^2^ = 0.065, CONF R^2^ = 0.118					
	Est	SE	Z	*p*	LLCI	ULCI
Direct effect						
CRE->PERF	0.08	0.06	1.55	0.122	−0.02	0.19
Indirect effect						
CRE->CONF->PERF	0.07	0.02	3.42	<0.001	0.03	0.12
Total effect						
CRE->PERF	0.16	0.05	3.00	0.003	0.06	0.26
Path coefficients						
CONF->PERF	0.22	0.06	3.92	<0.001	0.11	0.32
CRE->PERF	0.08	0.06	1.55	0.122	−0.02	0.19
CRE->CONF	0.34	0.05	6.99	<0.001	0.25	0.44
**COMP MEDIATION**	**PERF R^2^ = 0.055, COMP R^2^ = 0.096**					
	**Est**	**SE**	**Z**	***p***	**LLCI**	**ULCI**
Direct effect						
CRE->PERF	0.10	0.05	1.80	0.072	−0.01	0.21
Indirect effect						
CRE->COMP->PERF	0.06	0.02	2.98	0.003	0.02	0.09
Total effect						
CRE->PERF	0.16	0.05	2.94	0.003	0.05	0.26
Path coefficients						
COMP->PERF	0.18	0.05	3.40	<0.001	0.08	0.29
CRE->PERF	0.10	0.05	1.80	0.072	−0.01	0.21
CRE->COMP	0.31	0.05	6.23	<0.001	0.21	0.41

Note. CONF = Confidence; COMP = Competence; CRE = Creative self-efficacy; PERF = Perceived academic performance.

## Data Availability

The raw data supporting the conclusions of this article will be made available by the authors on request.
